# *Move:* Durational Dance-Making as We Age

**DOI:** 10.3389/fspor.2022.795526

**Published:** 2022-03-29

**Authors:** Jennifer Nikolai

**Affiliations:** School of Sport and Recreation, AUT University, Auckland, New Zealand

**Keywords:** screendance, care, dance and movement, aging, pandemic (COVID-19), practice-oriented research

## Abstract

Dance making and moving support a positive aging approach to aesthetic, cultural, and social meanings, thus, shaping dancers' lives. My perspective is informed by making dance a durational and sustained practice in the life of a long-life mover. Positioned as a practice-oriented researcher, I am surrounded by inspiring authors and artists who embrace maturity with improvisational making. This perspective piece reflects on making *Move*, a durational practice-oriented research process. *Move* contextualizes durational elements that I associates with the life of a mover; my writing and my companions improvising with my hand-held camera in isolation, in my neighborhood on Tāmaki Makaurau/Auckland's North Shore.This perspective piece opens a process I refer to as a *camera-dancer dyad*, a duet between a dancer and a camera, making in isolation as we age. Furthermore, by embracing the accessibility of everyday recording devices as dancing partners, dance making with mobile cameras becomes a playful partnership with a long life.

## Introduction

As proposed by Kontos (2005), the experimental approaches to dance-making can guide mature dancers through confidently critiquing the consumer culture rejection of the older body. Mature contemporary artists can access digital platforms, apps, and sites that now open opportunities to share or exhibit through synchronistic or asynchronistic platforms. In addition, by dancing with everyday recording devices such as the smartphone, we can create opportunities for compositional experimentation. When captured and reviewed through a camera, it is possible to share creative connections also, while we have limited changes to meet or travel and may feel isolated. Consequently, an accessible digital technology, when shared with the intention of care and connection, might bring our oceans together. In addition, the camera also helps us as performers to see in ways unavailable to us, while we improvise on our own, without partners or fellow cast members.

Compositional opportunities with a camera echo the experimental filmmaker Deren's essay, *Amateur versus Professional* (Deren, 1959), which accentuated that the camera relies on the body and an imaginative dancer, a choreo-cinema. Dance and/with/on a camera, as Deren explored foreshadows of Rosenberg's (2006) camera-looking, as a performance that, in itself, frames an event. My perspective is that, although we rely on technology to connect, we can utilize standard technology to enhance ways of sustaining the making of or the movement practice. I propose that the camera-looking opens a shared practice toward looking, with and as a result of forced limitations due to the COVID-19 pandemic.

Aligned with Kassel (2016), a dance and a camera disrupt a recognizable (theatrical) space, re-align gravity and ways of looking at making dance. “By using the camera, the possibilities of giving dance a form are extended” (p. 176–177). We connect with ways of moving, and ways of looking, with accessible hand-held recording devices. More significantly, we now access means of sharing online by sending a link or a clip to communities (of practice) we care about across the ocean(s). I propose that the camera-dancer dyad opens opportunities for us to keep moving and have dance-making with everyday technology as partners in a time of isolation. How can we curate with our mobile devices as we capture moments: shot, reviewed, re-shared based on what the mobile device captures? Without these devices, are we limited, without online, to shared events created at this time?

In this article, I explored the possibilities of the *camera-dancer dyad* as an everyday technology that enables sharing and connecting through dance. The *camera-dancer dyad* is a method of making dance while moving with the camera in the hands of the dancing camera operator. In the hand of the dancer, the camera takes compositional cues from that dancer, as opposed to a camera operator being external to the dancer's experience. As such, a *camera-dancer dyad* is a dyadic approach distinct from the tradition of a camera as an archival instrument. I argue, therefore, that the *pas de deux* between dancers and their cameras opens compositional opportunities for the dancing dyad as “instigator/provocateur” of mid-recorded improvisation (Nikolai, 2016, p. 131). From the position of the artist as a researcher, I propose a critical methodology, a *camera-dancer dyad* (the camera in the hands of the authoring dancer) that invites sustained practice(s) as a way of looking, inquiring, and making. With access to everyday technology in this time of adaptation, we can make in isolation and sustain future making/sharing.

Framing significant moments through the *camera-dancer dyad* opens the dance captured on mobile devices to non-linearity, a spreading of moments and sharing. In my work, I also engages improvised moments with a camera-dancer dyad. The compatibility of moving and making through a camera-dancer connection, I synonymously link to improvisation. The improvisation between the camera operator being the dancer, holding a recording device in the form of a camera opens compositional opportunities that may provoke further advocacy. This advocacy suggests that a mature artist carries a cumulative insight that ageism is re-evaluated toward propositions that dance is not *only* suitable for young performers (Nikolai and Markula, 2021). We can improvise with our cameras, be impromptu and responsive. This echoes what I read in Guazzanti (2021) regarding shooting a screen dance documentary; an unscripted or improvised movement occurs as an ethos of kindness, with the subject, human or non-human, partnering as a *camera-dancer dyad*. In this paper, I present my own experimentation with *camera-dancer dyad* partnering.

## The *Camera-Dancer Dyad* in Practice: On *Move*

During the Global Pandemic I was isolated from the more familiar process of improvising with fellow dancer. *Move* is a moving image study initiated on a shoal beach in the Waitematā Harbour, which is the Eastern access by sea to Aotearoa New Zealand, Tāmaki Makaurau/Auckland. *Move* as a piece/process developed while in isolation and continues as a durational study. Medium close-up and close-up shots of shadows contextualize improvisations with sand, seaweed and shells. My moving shadow(s) accumulate as my partner's light sources; the sun and tide pools.

*Move* consists of an accumulation of still images and moving image clips, all under 60s. Quiet opportunities with creatures of all kinds, non-humans living in tide pools, occupying spaces predicted by tide charts, with me. I find myself making these screen-dance “shorts” in an unlikely manner that I ever made previously. *Move* footage accumulates over all four seasons, initiated in the summer of 2020 and now into all seasons of 2021 and still to be played with into 2022. As I find myself making these “shorts,” I as a *camera dancer*, no one else informed me to direct my camera choices.

Living near the Pacific Ocean, the process of making *Move* began while improvising with the *camera dancer*, moving and shooting on a beach in my neighborhood. I took to improvising on a rocky shoal with the sand, the seaweed, and the saltwater at my feet. Isolated from other humans and creatures of all kinds became fellow partners. Inhabitants of my world (although being reduced from human-to-human contact) introduced me to the non-human. I resided within the vicinity of these creatures for so long, but I had not previously improvised in such a manner. Amidst the global pandemic, with a camera in my hand, the more-than-human now will continue to partner with my dance-making.

My storyboard that I had sketched on paper, flew from my hands into the mangroves. It then floated into the Pacific Ocean. When searching for the escaped storyboard, I spotted a blue surgical mask that attracted my attention, it resembled my paper storyboard, until I took a closer look.

Blue surgical masks have been washing ashore in 2020/2021, a previously unseen sight. The stranded storyboard and the surgical mask resided side by side until I found them. I took the storyboard and continued my process. This reminded me of Haraway's (2016) concept of tentacularity that is about a life lived along lines, and such a wealth of lines, not at points, not in spheres. Haraway continues: “The inhabitants of the world, creatures of all kinds, human and non-human, are wayfarers”; generations are like “a series of interlaced trails” (p. 2).

**Figure 1:**
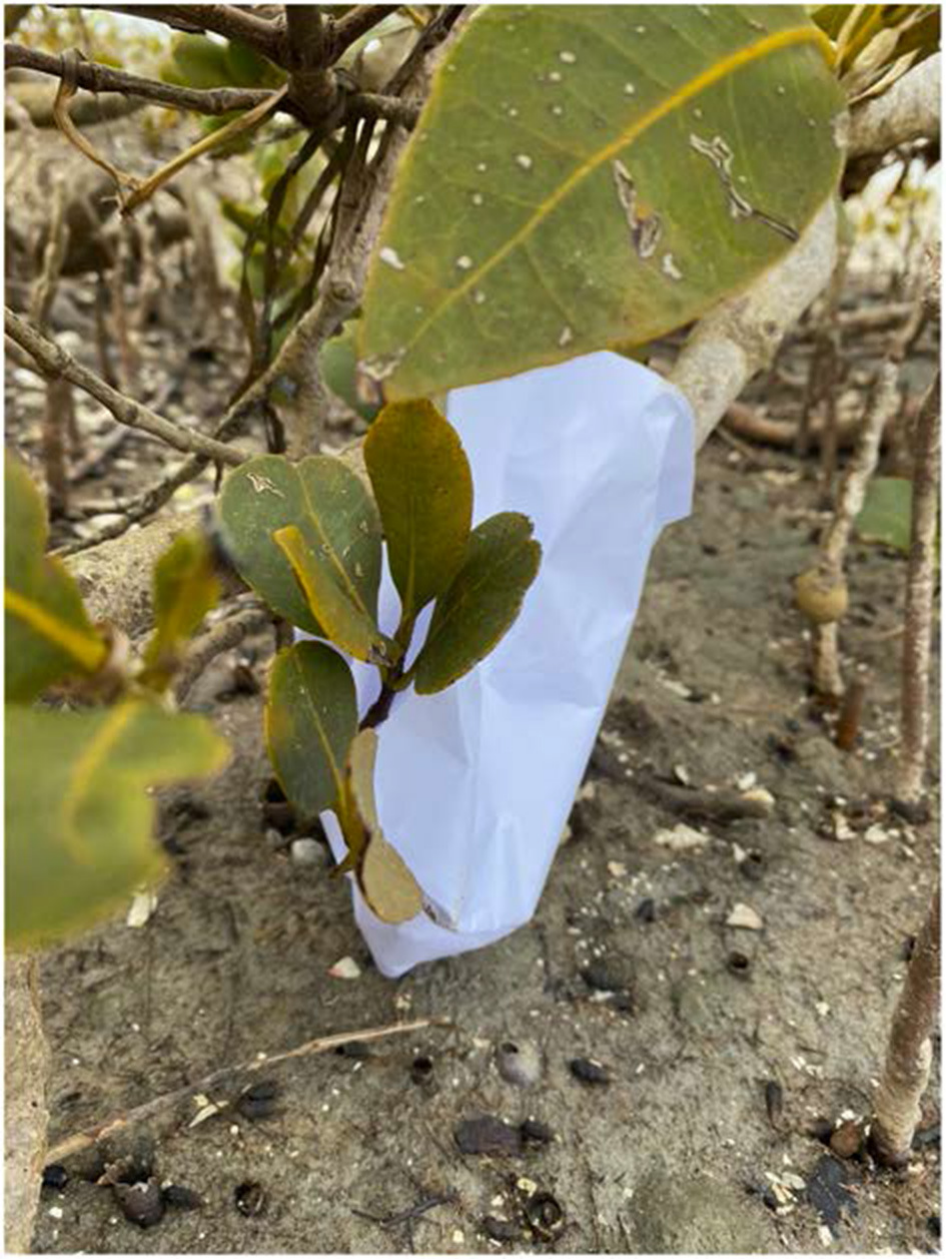
When the storyboard washed up among the mangroves in the Waitematā Harbour, also a Department of Conservation's domestic bird reserve, Tāmaki Makaurau/Auckland, Aotearoa New Zealand. December 2020. Photo credit, Jennifer Nikolai.

The tentacular nature of the surgical mask informed the improvisation that I played with while still holding my storyboard. It still echoes in the improvisational sentiments I make today. While the lines I live along are linked to seaweed and tide pools, the reflected surfaces I look into while shooting *Move* reflect my concern for our polluted oceans. The tide pools, once occupied with busy sea life, now contain garbage, newspapers, and more masks. I keep finding more masks (Figure 1).

**Figure 2:**
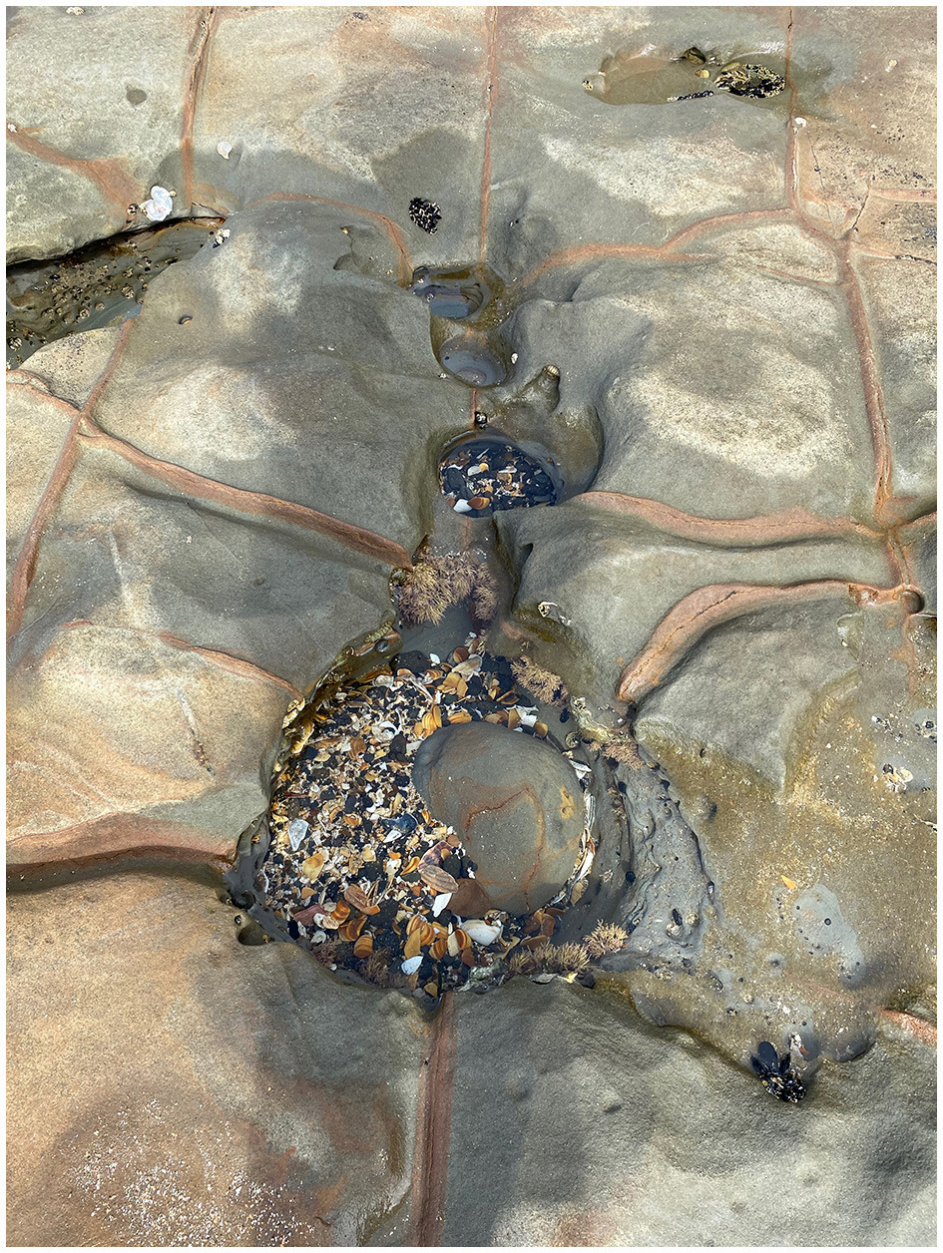
Shadow Tide Pool Trio. Waitematā Harbour, Takapuna Beach, Aotearoa New Zealand, September 2021. Photo credit, Jennifer Nikolai.

The dancers see ways of making and moving with improvised provocations guided by the dyadic act, of filming themselves. The dancers can make choices toward a shot type, framing themselves moving within the shot. The dancers can ultimately craft their compositional inquiry toward habitation with self, with other wayfarers—sand, seaweed, saltwater, shadows—partnered as a series of interlaced trails, caught improvising together (Figure 2).

In 2021, as more humans enter my space, I now duet with my distorted selfie, shooting my rippling shadow in a tide pool with a partner seaweed. We improvise with water, either still or crashing ashore. Longley's (2021) poetry echoes for me the care for improvisational partnerships with elements that are within my reach. The *camera-dancer dyad* collects what I improvise with “recognizing kindness as an ecological practice as we hold each other in our mattering, moved by the necessity of water (p. 281). *Move* carries characteristics of durational practice through making with the tides, with more than humans, but also within a global crisis. The eb and the flow of capturing screen-dance “shorts” as the years pass, as global circumstances shift constantly and urgently. The aging process is durational, echoing the moving image collection of sand, seaweed, self, as the tides come in and out.

Reflecting on my multiple years of making dance with cameras, I hypothesize that a camera in the hands of a moving dancer (*camera-dancer dyad*) aligns with Bergson's (1944) duration, as “the continuous progress of the past, which gnaws without ceasing, so also there is no limit to its preservation” (p. 173). Grosz (1999) identified that, with (Bergson's) duration as a whole, time is braided, intertwined, and unity of strands layered over each other: unique, singular, and individual. Duration, nevertheless, partakes of a more generic and overarching time, making possible relations of earlier and later, locating times and durations relative to each other (p. 17–18). Duration defies linear time, chronological time. Duration places its moments on either side of being “slackened and relaxed,” where what “these moments lose in reciprocal penetration they gain in respective spreading” (Deleuze, 1991, p. 86).

The attentiveness toward caring for oneself and others becomes heightened as our moments continue spreading. In my experience, moving becomes heightened as I age. The mobility of one day decreases the mobility the next; it frames the reflexive intention toward moving when we can, as we can, and with care.

## Author Contributions

The author confirms being the sole contributor of this work and has approved it for publication.

## Conflict of Interest

The author declares that the research was conducted in the absence of any commercial or financial relationships that could be construed as a potential conflict of interest.

## Publisher's Note

All claims expressed in this article are solely those of the authors and do not necessarily represent those of their affiliated organizations, or those of the publisher, the editors and the reviewers. Any product that may be evaluated in this article, or claim that may be made by its manufacturer, is not guaranteed or endorsed by the publisher.
